# Microgravity studies of solidification patterns in model transparent alloys onboard the International Space Station

**DOI:** 10.1038/s41526-023-00326-8

**Published:** 2023-10-18

**Authors:** S. Akamatsu, S. Bottin-Rousseau, V. T. Witusiewicz, U. Hecht, M. Plapp, A. Ludwig, J. Mogeritsch, M. Şerefoğlu, N. Bergeon, F. L. Mota, L. Sturz, G. Zimmermann, S. McFadden, W. Sillekens

**Affiliations:** 1 Sorbonne Université, CNRS-UMR 7588, Institut des NanoSciences de Paris, case courrier 840, 4 place Jussieu, 75252 Paris, Cedex 05 France; 2 Access e.V., Intzestr. 5, 52072 Aachen, Germany; 3 Laboratoire de Physique de la Matière Condensée, CNRS, Ecole Polytechnique, Institut Polytechnique de Paris, 91120 Palaiseau, France; 4 Department Metallurgy, University of Leoben, Franz-Josef-Str. 18, 8700 Leoben, Austria; 5 Department of Metallurgical and Materials Engineering, Marmara University, 34854 Maltepe, Istanbul, Turkey; 6 Aix Marseille Univ, Université de Toulon, CNRS, IM2NP Marseille, France; 7 School of Computing, Engineering, and Intelligent Systems, Ulster University, Northland Road, Derry/Londonderry, Northern Ireland BT48 7JL UK; 8 European Space Agency, ESTEC – Research and Utilisation Group, Directorate of Human and Robotic Exploration Programmes, Keplerlaan 1, 2201 AZ Noordwijk, Netherlands

**Keywords:** Materials science, Statistical physics, thermodynamics and nonlinear dynamics

## Abstract

We review recent in situ solidification experiments using nonfaceted model transparent alloys in science-in-microgravity facilities onboard the International Space Station (ISS), namely the Transparent Alloys (TA) apparatus and the Directional Solidification Insert of the DEvice for the study of Critical Liquids and Crystallization (DECLIC-DSI). These directional-solidification devices use innovative optical videomicroscopy imaging techniques to observe the spatiotemporal dynamics of solidification patterns in real time in large samples. In contrast to laboratory conditions on ground, microgravity guarantees the absence or a reduction of convective motion in the liquid, thus ensuring a purely diffusion-controlled growth of the crystalline solid(s). This makes it possible to perform a direct theoretical analysis of the formation process of solidification microstructures with comparisons to quantitative numerical simulations. Important questions that concern multiphase growth patterns in eutectic and peritectic alloys on the one hand and single-phased, cellular and dendritic structures on the other hand have been addressed, and unprecedented results have been obtained. Complex self-organizing phenomena during steady-state and transient coupled growth in eutectics and peritectics, interfacial-anisotropy effects in cellular arrays, and promising insights into the columnar-to-equiaxed transition are highlighted.

## Introduction

Solidification microstructures in alloys largely determine the properties of materials, and their characterization is of utmost interest in industrial research. It is common practice to identify those microstructures ex situ under the microscope and to measure their morphological features with full knowledge of the chemical nature of individual compounds and the physical properties of the mixture. Most often, however, information is lacking on the actual solidification path during the cooling process, and a clear interpretation of how the distribution of the microstructures in the bulk solid occurred cannot be achieved. The central question—how, upon cooling, a heterogeneous crystalline solid forms from a homogeneous liquid mixture—is actually strikingly complex when considered on a fundamental level^1,2^. Frozen-in or as-cast microstructures form out of equilibrium. They arise from self-organizing processes during growth at the advancing interface between the solid and the liquid. Some characteristic lengths and their approximate scaling with microscopic properties and control parameters can be derived theoretically. A scaling analysis is however insufficient: spatiotemporal phenomena during solidification depend on boundary conditions, initial conditions, and the whole history of the process^3^. Theoretical challenges are primarily associated with steady-state shapes and patterns, their morphological stability against symmetry breaking, and the formation of long-lived stacking defects within basically periodic arrangements. In addition, this dynamics can be influenced by instrumental characteristics and by the crystalline structure of the growing solid—being a single- or a polycrystal or made of crystals of different phases—via the so-called interfacial anisotropy. Such challenging issues call for fundamental research based on in situ experimental diagnostics, using systematic protocols guided by the general concepts of the nonlinear physics applied to solidification phenomena^4^.

In this review, we will report on recent observations made during microgravity solidification experiments using model transparent alloys. Focus is placed on unprecedented results obtained with two devices installed onboard the International Space Station (ISS), namely, the Transparent Alloys (TA) apparatus of the European Space Agency (ESA)^5^ and the Directional Solidification Insert of the DEvice for the study of Critical Liquids and Crystallization (DECLIC-DSI) developed by the French National Center for Space Studies (CNES)^6^ in close collaboration with NASA. Two distinctive features and strengths of this scientific research are worth underlining. First, considering the long characteristic times of the targeted phenomena, the ISS is the only facility that provides a stable reduced-gravity environment for experimental campaigns that typically require several weeks. Access is thus given to dynamic phenomena that are difficult to reproduce in a laboratory due, in particular, to the interaction of the solidification with convective motion in the liquid. High-quality experiments in essentially diffusion-controlled crystal growth conditions are key to a direct comparison with numerical simulations and an unequivocal identification of the relevant physical and geometrical parameters. Second, transparent alloys *that freeze like metals*^7^ have been used for many decades as model systems for in situ experimental studies of solidification patterns^8–12^. The shape of the solid growing from the liquid mixture can then be followed in real time over a large range of times and distances with flexible and technically light means of optical microscopy. Transparent-alloy solidification methods have evolved considerably over the last few decades^4,13–17^, thus strengthening their unique demonstration power. In parallel, the continual development of time-resolved numerical-simulation techniques has enabled increasingly direct, quantitative comparison of experimental observations with theory^18,19^. This synergy has already led to many new insights, with strong scientific impact in both materials science^1,2^ and the physics of nonequilibrium pattern formation^3,20^, and is also central to the scientific advances that will be reviewed here.

We will present first the scientific context of in situ solidification using transparent alloys, including the effect of convection in the liquid that may occur on ground. Second, the main innovative features of the TA and DECLIC-DSI apparatuses and the phase-field models for numerical simulations will be described. Third, we report on representative results on multiphased growth patterns in eutectic (lamellar-to-rod morphological transition, effect of a varying solidification velocity) and peritectic alloys (transient coupled growth) and on single-phased cellular and dendritic patterns (interfacial anisotropy, curvature of the isotherms, columnar/equiaxed transition). Finally, general conclusions and prospects are proposed in the “Outlook and Summary” section.

## Review

### Preliminary remarks

Most metals and some other compounds of various chemical nature, such as salts, rare gases, and some organic molecules, crystallize from the melt without faceting, in contrast to the vast majority of compounds^21^. On the atomic scale, nonfaceted crystals present a “rough” structure of the solid-liquid interface. The surface free energy (surface tension) of the solid-liquid interface is weakly anisotropic, and the shape of the crystals, both at equilibrium and during slow growth, remains smooth, without facets or sharp edges. The kinetics of attachment of atoms or molecules at the interface (interfacial kinetics) is relatively fast, and during growth, the interface can be considered locally at, or very near, equilibrium. Based on general statistical-physics arguments, a criterion was proposed by K. Jackson in the 1960s, which states that simply speaking, nonfaceted materials are such that their entropy of melting *ΔS*_*m*_ (per atom or molecule) is very close to, or not much greater than the Boltzmann constant *k*_*B*_^22^. Using that criterion, Jackson and Hunt found a series of nonfaceted materials made of small-sized, globular-shaped organic molecules that solidify from the liquid and form plastic-crystal phases^7^. The transparent compounds, such as succinonitrile (SCN), neopentylglycol (NPG), and d-camphor (DC), which are the focus of the present review, belong to this class of materials. The term “plastic” refers here to the large ductility of the molecular crystals in question. Plastic crystals present a high-symmetry (cubic or hexagonal) lattice with well-defined positional order but have a rotational disorder of the molecules^23,24^. Their crystal structure has been studied in various compounds, including SCN^25^ and carbon tetrabromide (CBr_4_)^26^, which are among the most common transparent analogs in solidification studies. Except for their lower thermal conductivity, nonfaceted organic compounds present physical properties relevant to solidification that are very similar to those of metals to within scale factors. This property justifies the use of low-melting nonfaceted organic alloys as excellent model systems for fundamental solidification studies.

Model alloys with low melting temperatures are particularly suitable for the implementation of automated solidification instruments intended for being installed onboard microgravity facilities^27–29^. There is a fundamental motivation for carrying out solidification studies in reduced gravity. The formation of remarkable shapes and patterns during solidification—generally speaking, during the growth of nonfaceted crystals from the melt—is primarily governed by the interplay of the moving solid-liquid interface with the diffusion fields of chemical species and heat in the liquid^1,2^. The diffusion gradients—the very ones that are responsible for the morphological instabilities of interest—create thermosolutal density gradients in the liquid. In the gravity field, the density gradients can induce convective flow, which perturbs the solidification dynamics on a large scale (see, e.g., refs. ^30,31^ and references therein; also see below). Microgravity in free-fall and orbiting facilities offers a unique way to suppress or drastically damp out thermosolutal convection, whatever the alloy composition and the geometry of the experiment (see, e.g., ref. ^31^). Solidification patterns can then be observed under purely diffusion-controlled growth conditions. The in situ experimental conditions and the corresponding observations can be more effectively analyzed with the help of theoretical and numerical models.

Diffusion-controlled crystal growth in nonfaceted alloys leads to various pattern formation phenomena, depending first on alloy characteristics. In a sufficiently dilute binary alloy a single-phased solid forms. For thermodynamic reasons, the solid and the liquid in contact do not have the same composition. This entails a redistribution of chemical species by diffusion in the liquid. A nonlinear coupling of the diffusion gradients with the shape of the advancing solid-liquid interface is responsible for the morphological instabilities of the growing solid. In contrast, the capillary response of the solid-liquid interface prevents strongly curved deformations of the solid-liquid interface, which is a stabilizing factor. The so-called cellular or Mullins-Sekerka instability of the planar front above a critical value V_c_ of the pulling velocity has been predicted on the theoretical basis just described^2^. By increasing V above V_c_, cellular patterns and dendritic arrays form, the space-time dynamics of which is sensitive to boundary and initial conditions. This complexity is common to a large class of nonequilibrium pattern-forming systems^3,20^. It motivates laboratory research involving well-calibrated in situ experiments and time-resolved numerical simulations that can be compared quantitatively with each other. This general comment also holds true in the case of eutectic and peritectic solidification, which presents the particularity to deliver a multiphased solid.

Large-scale solute redistribution gradients, along with temperature gradients, can trigger convective motion in the liquid phase during solidification. In ordinary conditions, natural convection in the melt perturbs both the temperature and solute concentration fields, which are no longer homogeneous on length scales on the order of the size of the container. This entails a substantial modification of the microstructure. This has been evidenced, in particular, by comparing experiments performed with the DECLIC-DSI apparatus on-orbit on the one hand and with a twin apparatus on-ground on the other hand^32–34^: convection can induce the appearance of localized patterns and superstructures or modify the primary cell spacing distribution. Interestingly, forced or enhanced convection by centrifugal motion^35^ and electromagnetic forces^36^ (also see ref. ^37^) have been shown to trigger phase separation or influence microstructural transitions. These effects are naturally absent in the microgravity conditions existing in solidification facilities onboard the ISS.

### Methods and apparatuses

The multi-user TA program encompasses the study of (1) steady-state and transient multiphased growth patterns in eutectic and peritectic alloys and (2) the columnar-to-equiaxed transition in single-phased alloys. The DECLIC-DSI setup was designed to study the spatiotemporal dynamics of cellular and dendritic arrays. Both apparatus (as well as the numerical simulations) use the directional-solidification configuration (also referred to as the Bridgman method), during which the sample is displaced at an imposed velocity V in a fixed temperature field with a controlled axial temperature gradient G (axis **z**). In the experiments, the displacement (also called “pulling”) velocity V typically ranges from 0.010 to 100 µms^−1^, and G between 10 and 80 Kcm^−1^, depending on the system under study. The pulling velocity V is the principal experimentally accessible control parameter. In steady-state, the solidification rate is equal to V, and the solid-liquid interface stays, on average, at a constant position in the temperature field. Ideally, the isotherms remain flat, perpendicular to the pulling axis **z**, and stationary in the laboratory frame. In many cases, this “frozen-temperature” assumption is essentially valid. In other cases, the motion or a distortion of the gradient field must be considered, as will be mentioned below. In the experiments, the solidified alloys are contained within transparent, glass-wall cartridges. That the position of the solidification front is essentially constant in the laboratory frame allows continuous observation with a fixed optics. More detailed characteristics specific to the TA and DECLIC-DSI instruments, respectively, as well as the phase-field numerical-simulation method, are presented below.

**Fig. 1:**
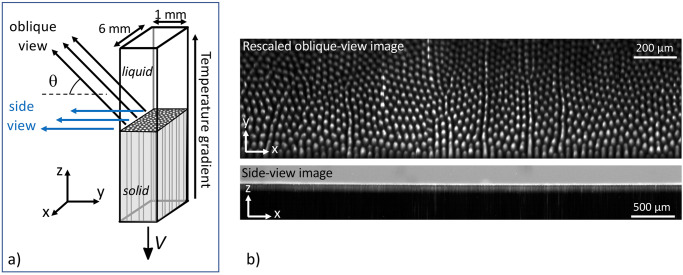
The DIRSOL method in the TA apparatus. **a** Schematic representation. V: pulling velocity. θ: viewing angle. **b** In situ TA images during directional solidification of a eutectic SCN-DC alloy (V = 0.007 µms^−1^). Top: detail of an oblique-view image (rescaled to 1:1 aspect ratio) of a rod-like eutectic pattern, with DC rods appearing white and the SCN matrix dark^59^. Bottom: side-view image (liquid on top, eutectic solid on bottom).

The design of the (multi-user) TA apparatus, developed by QinetiQ Space (now Redwire Space), was based on the DIRSOL method previously developed at INSP (Fig. 1a)^15^. Its principle is derived from the conventional thin-sample directional solidification technique. Flat-wall (fused silica) samples with a rectangular cross-section (6 × 1 mm^2^ in the case of TA eutectic-alloy cartridges) and a length of several cm are filled under a protective Ar atmosphere with the molten alloy prepared with purified compounds and sealed. For certain applications, a crystal selector at the cold end is used to grow a single eutectic grain, except for a few sub-boundaries. In the apparatus, the temperature gradient (G = 30–60 Kcm^−1^) is established between two metallic blocks separated by a 7-mm gap (also known as the adiabatic zone in the scientific literature). Each of those blocks is made of two pieces (also called clamps) with independent thermal regulation and assumed to have good thermal contact with the cartridge walls.

The observation is made from the outside, in oblique incidence with respect to the solidification axis. Large working distance optics with a small, tunable numerical aperture is used under dark-field observation mode (Fig. 1b). In the TA apparatus, the inclination angle θ of the optics is fixed to a value (43.6°) close to an optimum value such that a sharp image of the entire pattern is obtained (the imaging plane coincides with the image of the growth front through the liquid and the glass). The small aperture and a finely tuned inclination of the transmitted-light illumination make it possible to select rays coming from one of the two solid phases only^15^. This also limits the astigmatism due to the plane diopters—the TA optics includes an additional astigmatism correction device for optimizing the sharpness of the images. The effective resolution of about 3 µm is sufficient to study the overall dynamics of the structure. A numerical camera is coupled to the optics for real-time imaging. The TA setup also possesses a second camera for side-view observations (Fig. 1b). During eutectic growth experiments, this observation mode was used to follow the average position of the solidification front and to monitor its shape (essentially planar in Fig. 1b) along the *x*-axis. The side-view imaging was the main mode of observation for peritectic and columnar/equiaxed structures.

The TA apparatus was launched to the ISS in December 2017 in the first CRS (Commercial Resupply Services) flight of the SpaceX Dragon using a Falcon 9 launcher whose first stage was reused. It was installed in the Microgravity Science Glovebox (MSG) facility onboard the ISS in January 2018. The astronauts were in charge of the introduction of the cartridges into TA, in addition to the installation of the apparatus in MSG. The major parts of the operations are essentially automatic, with telescience (telemetry control of parameters, image frequency, etc.) used from the operation center (E-USOC, Madrid, Spain) permitting near real-time feedback. Three TA cartridges of SCN-DC-based eutectic alloys were used successfully onboard the ISS. Two of them were processed during the SEBA (Solidification along a Eutectic Path in Binary Alloys) campaign and a third one during the SETA (Solidification along a Eutectic Path in Ternary Alloys) campaign. Peritectic-alloy cartridges have been used for the METCOMP (Metastable Solidification of Composites: Novel Peritectic Structures and In-Situ Composites) program. Some preliminary results obtained during the CETSOL (Columnar to Equiaxed Transition during SOLidification processes) campaign will also be presented. It can be noted that SETA/SEBA, CETSOL and METCOMP were former MAP (Microgravity Application Promotion) programs of ESA. The TA results reported here have been obtained during TA campaigns spanning between the years 2018 and 2022.

The DECLIC facility is a compact, multi-user facility for conducting experiments in the fields of fluid physics and materials science, and, more generally, on transparent media within the ISS environment. The main part of DECLIC is common to all experiments and contains electronics (for regulation, data acquisition and management, communication, etc.) and some optical resources (laser, optics, cameras). Different inserts that contain elements specific to each experiment complete the description of the facility. The DSI is the insert dedicated to solidification studies to image the dynamics of cellular and dendritic arrays—for a detailed description, see ref. ^38^. In the DECLIC-DSI, a cylindrical, quartz-wall cartridge (inner diameter of 1 cm; typically with a solidification length of 10 cm) is inserted in a Bridgman furnace (V = 0.1 to 30 µms^−1^). A system of volume compensation accommodates the density variations associated with phase changes in the alloy. This configuration permits the study of extended patterns from initial stages up to steady-state growth conditions being established. Temperature gradients G ranging from 10 to 30 Kcm^−1^ are imposed between the hot and cold zones^6,39^.

**Fig. 2:**
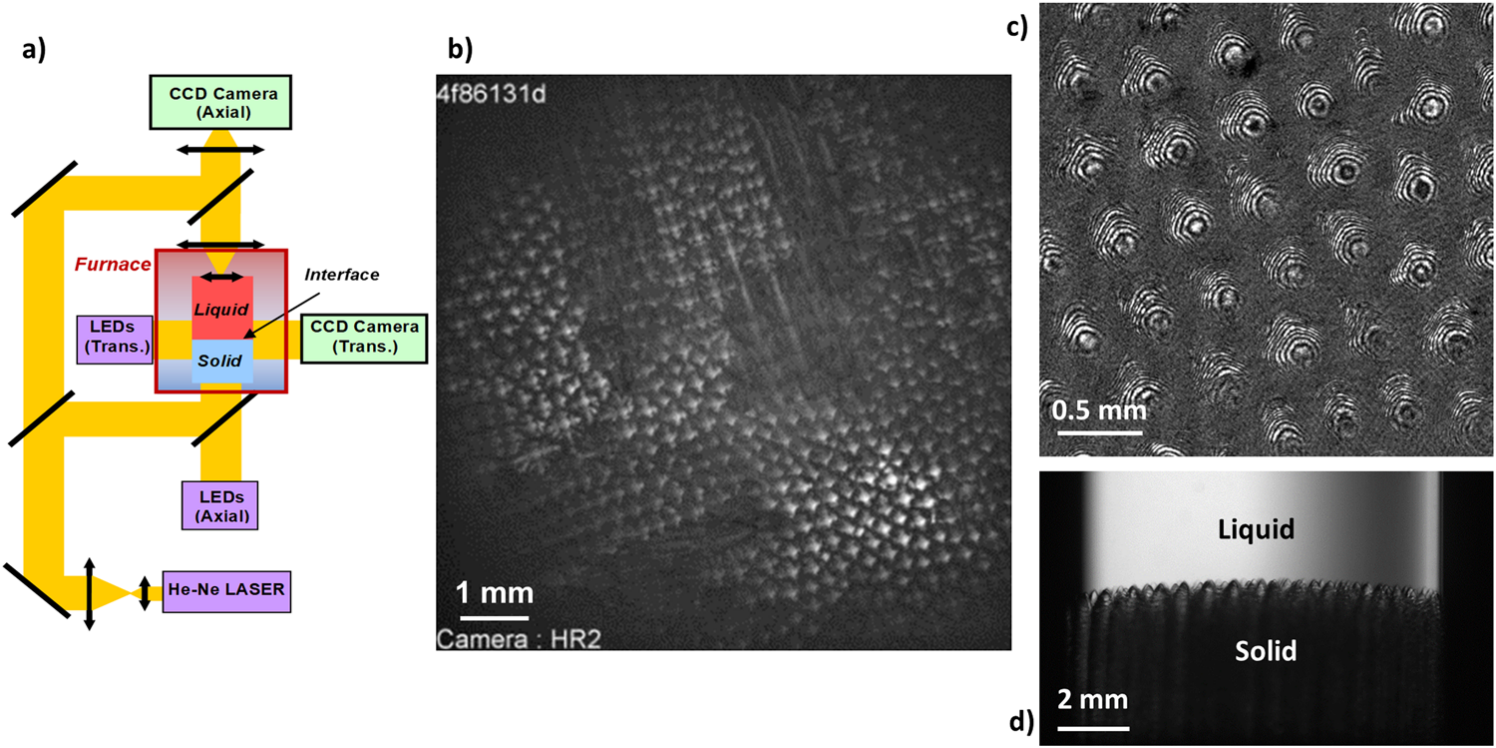
The DECLIC-DSI. **a** Optical diagnostics (schematics). Examples of images using the different diagnostics (SCN-0.46 wt%DC alloy; G = 12 Kcm^−1^; microgravity experiments). **b** Top-view image of a dendritic pattern (V = 8 µms^−1^); **c** Interferometric observation of a dendritic pattern (V = 1 µms^−1^); **d** Side-view observation (V = 1.5 µms^−1^). Color online.

A schematic of the optical diagnostics is given in Fig. 2a. The crucible is equipped with a circular, optically flat glass window at the bottom and a lens immersed in the melt at the top. The axial observation mode takes advantage of the complete transparency of the experimental cartridge. The light coming from the axial LEDs is transmitted through the solid and the liquid, crossing the interface, the image of which is formed on a CCD camera. On the same axis, a Mach-Zehnder interferometer (He-Ne laser) produces interferometric images used for high-resolution, three-dimensional shape reconstruction^14,40^. Additionally, transverse observation optics provides side-view images of the interface on a millimeter scale^39^.

The main instrument monitoring was operated from the CADMOS center, the French User Support and Operation Centre (USOC) in Toulouse, France. Taking advantage of the provided telescience capabilities, scientists were given the possibility to follow the solidification pattern in near real-time conditions and to remotely control the experiments.

For about 20 years, the phase-field method has become the method of choice for modeling and simulation of solidification, crystal growth, and microstructure evolution^41–45^. Its advantage is that it avoids front tracking by representing interfaces as steep but smooth (i.e., diffuse) variations of one or several scalar fields, which are the so-called phase fields. The equations of motion for the phase fields are coupled to the transport equations for all relevant quantities (heat, solute, momentum) following the principles of out-of-equilibrium thermodynamics.

A major step forward in the applications of phase-field models for solidification was the development of the thin-interface limit for pure substances^45^, binary alloys^46,47^ and eutectic alloys^48^. With this method, it is possible to perform quantitative (accurate) simulations using a thickness of the diffuse interfaces that can be orders of magnitude larger than the physically realistic value. The associated gains in computational performance have made it possible to perform realistic simulations, both in two and three dimensions, on the scale of the microstructure.

To perform the simulations, in-house codes are commonly written in C or FORTRAN, and modern methods are used for computational speedup (preconditioning, GPU parallelization, multi-grid methods). These codes typically run on computational clusters on the laboratory scale (a few GPUs or a few hundred CPU cores) within processing timeframes of hours to days. This allows for a detailed and direct confrontation between experiments and simulations.

### Eutectic growth

Eutectic alloys present the specific case of solidifying into a multiphase solid with self-organized composite microstructures^2^. In a binary eutectic alloy, the liquid and two distinct solid phases, generically noted as α and β, can coexist at equilibrium at the (unique) eutectic temperature T_E_. Their respective concentrations C_E_, C_α_ and C_β_ are fixed (C_α_ < C_E_ < C_β_). During directional solidification, the two solids can grow simultaneously and form quasi-planar, two-phased solidification patterns. The coupled-growth dynamics is mainly governed by solute diffusion in the liquid on the scale of the (interphase) spacing λ, and capillary effects. In steady-state, coupled-growth patterns basically exhibit banded or hexagonal arrangements, thus delivering lamellar or rod-like microstructures in the solid. As shown by Jackson and Hunt in the 1960s, the average spacing remains close to a characteristic length λ_JH_, which varies as V^−1/2^ with the pulling velocity V^9,10^. Rod-like and lamellar patterns are morphologically stable for λ values ranging within finite intervals, the limits of which approximately follow the same V^−1/2^ scaling as λ_JH_^49^. Outside their stability domains^50^, lamellar and rod-like patterns undergo secondary, symmetry-breaking instabilities and complex spatiotemporal phenomena^51,52^. Anisotropic behavior due to crystal orientation effects in eutectic alloys has also been carefully studied^17,53,54^. In the current review, we assume an essentially isotropic system.

Space experiments permit the study of the morphological stability of lamellar, rod-like, or more complicated patterns and their dependence on the alloy concentration C_0_ ranging over a finite interval about C_E_^55,56^. For an off-eutectic alloy (C_0_ ≠ C_E_), there exists a diffusion layer of extension l_d_ = D/V (with D being the solute diffusion coefficient in the liquid), which can reach several millimeters for solidification velocities below 1 µms^−1^. That layer, which superimposes the λ-scale modulation of the diffusion field, has little influence on the coupled-growth dynamics but can trigger convection in the liquid. The composition and thermal fields in the liquid are then perturbed on a large scale, and vastly different morphologies can be seen simultaneously in the same sample^57^.

Two SEBA cartridges and one SETA cartridge were used successfully with the TA apparatus onboard the ISS. The two main questions that were addressed are described as follows. The first question (SEBA) concerns the so-called lamellar-to-rod transition: in simple terms, in a given system, is it possible to induce a morphological transformation of rods into lamellae and/or the reverse by changing the pulling velocity? The second question (SETA) relates to the response of rod-like patterns to an imposed variation of the pulling velocity and whether there exists a spacing and/or order selection mechanism induced by such temporal “velocity ramps”.

#### The first study presented here concerns the lamellar-to-rod transition in binary eutectics

Eutectic solidification microstructures are often either of the rod-like or the lamellar type. Lamellar patterns prevail in alloys with nearly equal phase fractions (η ≈ 0.5, where η is the volume fraction of the β phase), while rod-like patterns are observed for markedly departing phase fractions (typically η < 0.25). Due to mass conservation, η is determined by the lever rule in the phase diagram^10^. A lamellar-rod transition is thus expected to occur in a given system upon varying the concentration (and hence varying η). This has been essentially confirmed experimentally^57^ and numerically^58^, but complex, unsteady dynamic processes involving hybrid shapes were mostly revealed.

When turning attention to an alloy with fixed composition, it has been shown numerically that rod-like and lamellar stability intervals overlap with each other. Within the bistable region of parameters, a coexistence between well-ordered lamellar and rod-like patterns is shown to be possible. In addition, numerical simulations suggest that the transformation of lamellae to rods occurs in a propagative way. As reported shortly, the main observations made during the first SEBA campaign confirmed this scheme and brought additional pieces of information^59^.

Rod-like patterns in the SCN-DC eutectic system have been studied previously on ground using the DIRSOL technique^60–62^. Systematic experiments consisting of observing the response of steady-state patterns to stepwise, downward or upward changes of V were used to identify the upper and lower stability limits of hexagon patterns (rod splitting and rod elimination instability thresholds, respectively)^62^. Observations made during the SEBA campaign^59^ were in full agreement with previous conclusions—the high-level performance of the TA apparatus was also attested (see Fig. 1b). In practice, a stretching of the pattern (thus an increase of λ) in the transverse direction was also observed, both during on-ground and space experiments. This phenomenon was due to a mild curvature of the isotherms, as imposed by different thermal conductivities of the cartridge walls and the alloy. At long times, the increase of the spacing was counterbalanced by rod splittings, and the system converged toward a quasi-steady spacing distribution (with λ_av_ ≈ λ_JH_)^61^.

**Fig. 3:**
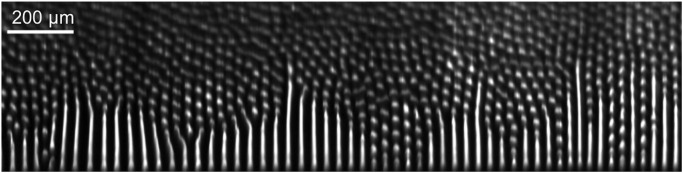
Lamellar-to-rod transition observed in situ. Directional solidification of a eutectic SCN-DC alloy with tilted isotherms (V = 0.007 µm s^−1^) in microgravity (TA apparatus)^59^.

In order to observe straight lamellae and their mode of transformation into rods on long timescales, a mild transverse component was imposed on the temperature gradient. This was made possible by the four hot- and cold-clamp temperatures being regulated independently. The isotherms were inclined by an angle of about 6.3 ± 0.3°. This entailed a global drift of the coupled-growth pattern in the direction perpendicular to the sample walls^63^. A large lamellar domain was observed to grow (Fig. 3). The lamellae were stabilized in contact with the sample wall. At the free ends, the lamellae underwent a breakup instability and emitted new rods in a loosely regular way. The process was propagative, but in the configuration with tilted isotherms, the breakup instability was slower than the geometrical drift of the pattern. This permitted the extension of a stable lamellar domain. A long-lived coexistence of straight lamellae and rods separated by a sharp domain wall was thus evidenced. There was strong evidence that, at low velocity, a lamellar pattern of infinite extension would remain absolutely stable^64^.

**Fig. 4:**
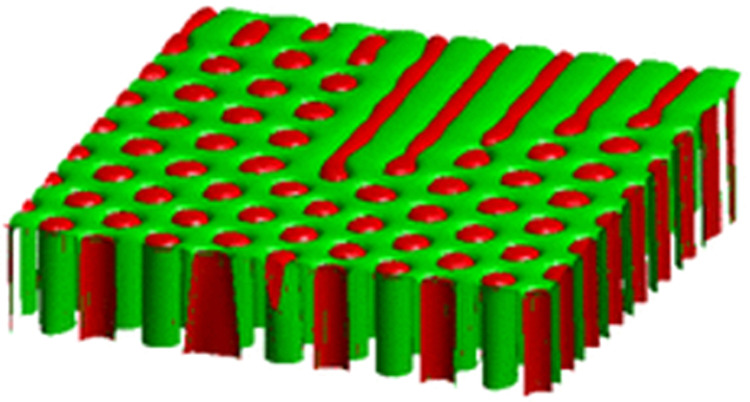
Lamellar-to-rod transition in a numerical simulation. Three-dimensional phase-field simulation of a model alloy with symmetric phase diagram and volume fraction η = 0.34. The tilt of the isotherms with respect to the pulling direction is 4°. Zero-gradient boundary conditions are applied to phase and concentration fields at the front and rear (corresponding to the presence of the glass plates in the experiments) and periodic boundary conditions on the sides. Lamella form at the rear side and slowly elongate, while pinchoff of new rods occurs periodically at the lamella terminations. Color online.

This scenario was tested by three-dimensional phase-field simulations with the model that was developed in ref. ^48^ and used previously to study the stability of rods^58^ and lamellae^65^, as well as the effect of a transverse temperature gradient^63^. A model phase diagram that was completely symmetric with respect to the exchange of the two solid phases was used rather than the SCN-DC phase diagram in order to maximize computational efficiency. An example of a simulation with a tilt angle of 4° that was started from an initial condition with a random distribution of α and β phases in the solid is shown in Fig. 4. As in the experiments, lamellae start to form on one side of the system and slowly propagate toward the other side, while they are shortened by the pinchoff of new rods at the lamella terminations. A systematic variation of the alloy concentration, pulling velocity, and tilt angle yielded the following main results. (1) For a given velocity and tilt angle, lamellae propagate across the system above a critical volume fraction (or concentration), below which only rods are present. This critical composition depends on V. (2) For a given composition and tilt angle, lamellae are formed below a critical pulling velocity which depends on the composition. (3) The critical composition or velocity also depends on the tilt angle. These results are fully consistent with the experimental observation and demonstrate that this mechanism of the rod-to-lamella transition is generic and not limited to the SCN-DC alloys system.

There are still many pending questions, in particular concerning the precursory instability mode, which resembles a Rayleigh-Plateau instability of a liquid jet, and the fact that the stability of the lamellae does not follow the ordinary V^−1/2^ scaling. Finally, additional observations were made in a second SCN-DC cartridge in the TA apparatus, following a nearly similar experimental protocol but without using the crystal selector. Several eutectic grains were present during solidification, and smooth but clear crystallographic effects were observed. Ongoing numerical simulations are expected to bring more detailed information on those questions.

#### The second study concerns the dynamics of eutectic growth under imposed velocity transients

Rod-like patterns with local hexagonal order most commonly contain a large number of topological defects, mostly heptagon/pentagon pairs in chain-like arrangement delimiting small hexagon domains. The SETA experimental program was inspired first by an observation reported in an Al-Al_3_Ni eutectic alloy^66^, bringing experimental evidence that the degree of order (or disorder) of rod-like eutectic patterns can differ strongly depending on whether a given V value is reached upon either increasing or decreasing the pulling velocity. A similar conclusion was drawn in ref. ^58^, as well as in a recent phase-field simulation study^67^. However, by comparing those studies, it appears that the way order or disorder is reached depends strongly on the system and the geometry of the experiment. In brief, a practical but complex question was to determine whether transient dynamics during a solidification experiment with a controlled temporal variation of the pulling rate can lead to a rod-like pattern with improved quality, that is, a better organized hexagonal arrangement.

**Fig. 5:**
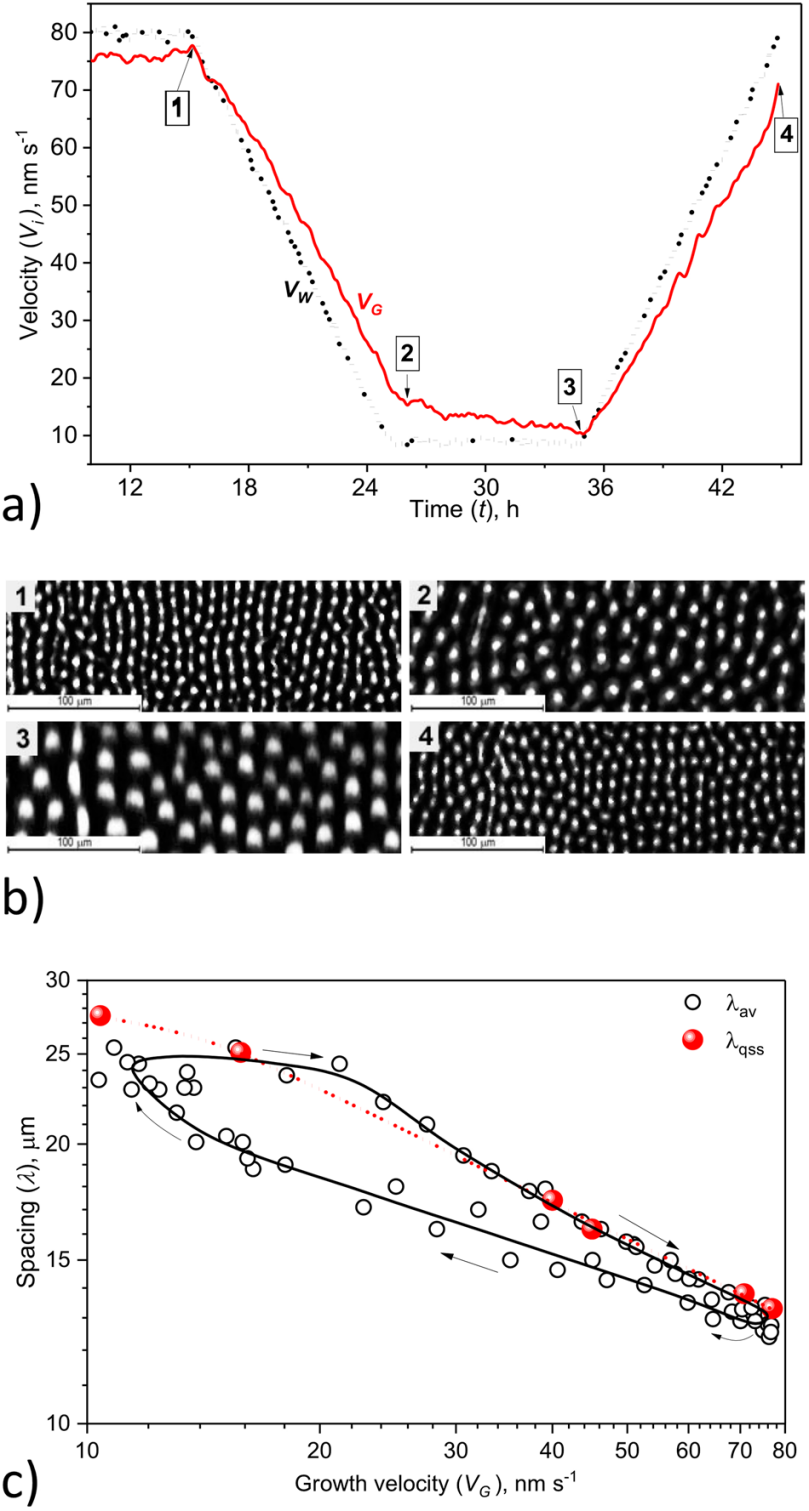
Rod-like eutectic growth patterns with varied solidification velocity. Directional-solidification of a eutectic SCN-DC alloy with a third compound (NPG) in microgravity (TA apparatus). **a** Nominal (dots) and actual (red line) growth velocity as a function of time. **b** Images (details) of the rod-like eutectic structure (see labels in **a**). **c** Average rod spacing λ_av_ as a function of the actual growth velocity during the same experiment (black dots; the black line is a guide for the eye). Arrows: time variation. Large red disks: average spacing λ_qss_ measured during (separate) quasi-steady experiments. Red dotted line: λ_JH_. Color online.

A series of transient solidification experiments with different acceleration and deceleration profiles were performed on ground and onboard the ISS^68^ using a binary eutectic SCN-DC alloy and a univariant (two-phase) ternary eutectic SCN-DC alloy with a small amount (0.5 wt%) of the third compound (NPG)^69^. Positive or negative (temporal) V-ramps, that is, accelerated or decelerated V(t) profiles with stepwise, linear and exponential variations, were imposed. This made a huge number of observations that are currently under analysis, mainly in terms of the evolution of the rod-spacing distribution (histogram) and the degree of hexagonal order. The following conclusions have been tentatively drawn—they are illustrated in Fig. 5.

Upon decelerated growth, the average spacing λ_av_ increases, as expected from the approximate λ_JH_ scaling. However, the spacing adjustment is slow and lags behind the scaling law predictions, which is primarily due to the increasingly slow dynamics of the rod elimination process. The slow-V, large-λ pattern obtained at the end of the decelerating stage experiences a re-acceleration phase without having established a stable steady-state distribution. Upon accelerated growth, λ_av_ decreases, and the spacing evolution nearly follows the scaling law predictions, which is due to the fast rod-splitting dynamics. It appears thus that an imposed V(t)-profile program with alternatingly negative and positive V-ramps results in hysteresis-like dynamics that can be clearly visualized in a λ_av_(V) diagram. The width of the hysteresis loop and also the improvement (or degradation) of the hexagonal order depend on the shape of the V(t)-profile.

### Peritectic growth

Peritectics are found in many industrial alloys such as steels and other Fe-based alloys, Cu- and Ti-alloys, some magnetic materials, and superconductor alloy^70^. A peritectic transition involves a liquid and two solid phases, which coexist at the peritectic temperature T_p_, the concentration of the liquid (C_L_), that of the primary phase α (C_α_) and that of the secondary (also called peritectic) phase β (C_β_, or C_p_) being such that C_α_ < C_p_ < C_L_. The peritectic growth dynamics depends strongly on whether the average concentration of the alloy C_0_ is larger or smaller than C_p_ and on experimental conditions.

During directional solidification, the primary phase commonly grows with dendritic morphology, and the peritectic phase β forms at the rear of the dendrite tips, in contact with the liquid at the peritectic temperature T_p_ (peritectic reaction). There is also a strong driving force for the secondary phase to continue to grow below T_p_ so that the primary phase can be eventually absent from the final bulk microstructure.

For solidification velocities that are slow enough to avoid cellular instability, the primary solid initially grows with a planar α-liquid interface at a temperature that continuously decreases with time due to solute redistribution. For temperatures below T_p_, the liquid becomes metastable against the nucleation of the peritectic phase β. For a hyper-peritectic alloy (C_p_ < C_0_ < C_L_), this may lead to a steady-state growth of the β phase. For hypo-peritectic alloys (C_α_ < C_0_ < C_p_), no stable conditions for planar growth of single-phased—either α or β—solids exist. Two complex solidification regimes have been observed and analyzed in previous studies. In the first regime, thin α and β crystals grow laterally and form thin bands that alternate in the direction of the temperature gradient (banded growth; see ref. ^71^ and references therein). In the second regime, the two solid phases grow simultaneously, thus forming peritectic coupled-growth (PCG) microstructures that resemble regular eutectic ones. Such PCG patterns were found to depend on various factors (see, e.g., refs. ^72,73^), including convection ahead of the front^74,75^. To suppress the latter, a first microgravity investigation of PCG was performed in hypo-peritectic Cu-Sn alloys onboard the ISS (M. Rappaz, private communication). Indeed, PCG microstructures were found, but the presence of large gas pores—one of the most prohibitive disturbances during solidification studies^76^—made the analysis difficult, if possible at all.

Besides the requirement that the primary phase is morphologically stable, little is known about the conditions under which PCG is possible and about the transient that leads to its emergence. Experiments on a transparent peritectic system under microgravity offer an unprecedented possibility to study these questions further.

The transparent organic peritectic model system Tris(hydroxymethyl)aminomethane-Neopentylglycol (TRIS-NPG) was used for in situ microgravity studies (METCOMP program) in the TA apparatus^77,78^. Three TRIS-NPG alloys of nominal concentrations (50, 52, and 53 mol%NPG, respectively) close to the peritectic concentration C_p_ have been processed and 18 individual solidification experiments were realized. A typical experiment was performed (after a 2–3 h of annealing time) at constant velocity (V = 0.04–0.18 µms^−1^) in a temperature gradient G of about 40 ± 10 Kcm^−1^. The solid-liquid interface was observed in side view. Thanks to a slight transverse temperature gradient, the structure of the solidification front could be imaged by using focus series. The challenge for studying PCG patterns in the TRIS-NPG system is that the primary and peritectic phases have nearly equal refraction indices. As indicated below, the distinction between the two phases could nevertheless be made by observing their different dynamical behaviors.

**Fig. 6:**
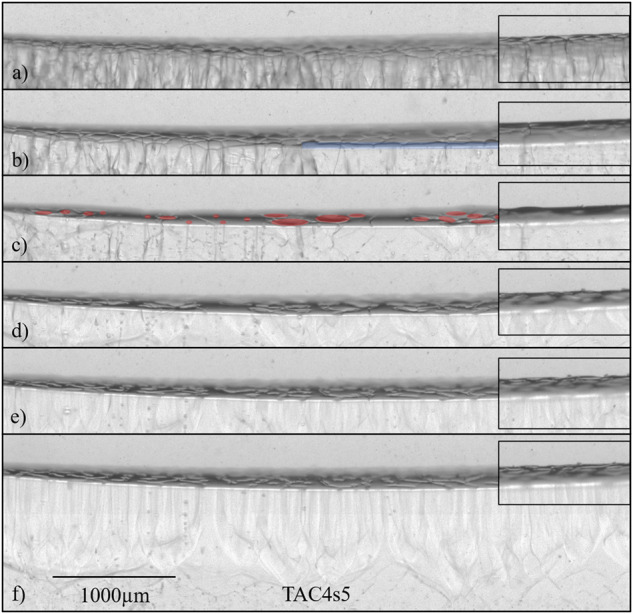
Peritectic coupled growth. Formation of PCG during directional solidification of a near-peritectic TRIS-51 mol%NPG alloy in microgravity (TA instrument, ISS). The side-view images were taken with middle focus except for the insets, which were taken with front focus. The photograph in (**a**) was taken 1.5 h after a pulling velocity of 0.11 µms^−1^ was activated. The snapshots in panels (**a**–**f**) were evenly spaced in time, with a time interval of 1 h.

During the initial annealing stages (V = 0), two processes occurred simultaneously: (1) the grains of the initial (primary-phase) polycrystal coarsened, and (2) inclined grain boundaries migrated toward the solid-liquid interface in the direction of the gradient axis (temperature gradient zone melting or TGZM process^1,2,79^). Figure 6 shows an example of how PCG formed during solidification. At the moment the pulling velocity was set to V = 0.11 µms^−1^, the α polycrystal was made of elongated grains (as in Fig. 6a). After a certain time of pulling, an elongated crystal (blue band in Fig. 6b) was observed to grow close to the back wall. We could get evidence that this new crystal appeared at the interface at a time when the temperature of the front was below the peritectic temperature and was identified as being of the peritectic phase β. It propagated in the thickness of the sample while leaving some α crystals growing simultaneously (red patches in Fig. 6c). In other words, the two (primary and peritectic) phases were observed to grow in a coupled way. This is, to the best of our knowledge, the first real-time observation of a transient solidification regime leading to PCG.

An interesting morphological evolution from large rods to lamellae (possibly drifting or with a zigzag shape) was observed (Fig. 6c–f). This occurred while the front was recoiling toward lower temperatures along the temperature gradient axis. The change of the microstructure in the solid can also be noted in the last image (Fig. 6f). During the following stages (not shown), a decrease of the α phase fraction was observed until complete disappearance. Thus, in brief, during the recoil of the solidification front, we observed a transition from α-phase to β-phase growth via a transient α-β PCG regime. A more accurate analysis of the recoil of the front still remains to be made. It obviously corresponds to an unsteady solute redistribution process, which might involve impurity pile-up, a plausible large-scale composition gradient in the cartridge, and a possibly temperature-dependent solute diffusion coefficient in the liquid^80^. Nevertheless, the actual solidification dynamics was favorable to the occurrence of a PCG sequence in the course of a long-lasting recoil of the solidification front.

The front recoil complicates the theoretical analysis of PCG. In fact, similarly to what was observed in ground experiments on Cu-Zn^81,82^, PCG is initiated before the solute boundary layer has fully built up. Further evolution of this layer over time therefore creates growth conditions for the coupled-growth structure that vary on a slow time scale and ultimately leads to the termination of PCG in the present experiments. This non-steady nature is a general feature of PCG in alloys with large solidification intervals. Regarding the initiation of coupled growth, in comparison to eutectic coupled growth, there are both similarities and differences. In both cases, coupled growth is initiated starting from single-phase growth when the second phase emerges at the front, either by nucleation or by growth from a seed deep within the solid. In eutectics, coupled growth emerges either by the instability of a finger of the second phase that propagates along the front^83^ or by the immediate emergence of a two-phase pattern^84^, and the initial spreading of the two-phase pattern is followed by coarsening. For the transparent peritectic system studied here, the dynamics of initiation is rather slow, and the structure refines rather than coarsens after its emergence. This mechanism for the emergence of peritectic growth is very different from the successive nucleation and overgrowth that has been postulated for Fe-Ni in previous works^85^.

To make further progress, the dynamics of the transient leading to PCG needs to be studied in a more systematic way. This will certainly also shed new light on the question of which alloys and under which conditions PCG can occur. Three-dimensional phase-field simulations of peritectic growth would be very useful to supplement the experiments.

### Cellular and dendritic growth

In directional solidification of a (semi) dilute alloy, increasing the pulling velocity above the threshold value V_c_ leads to a morphological instability of the planar, single-phased interface^1,2^. As mentioned above, a fingering, cellular microstructure first develops for V not too far above V_c_. For higher V values, deep cells undergo a sidebranching instability and evolve toward a dendritic array pattern. The growth dynamics of both cellular and dendritic arrays is complex and involve various length scales that are to be characterized. It is common to start with the most evident pattern characteristic size, namely the primary spacing λ, which corresponds to the distance of repetition in a periodic structure, and to proceed with the analysis of sidebranching characteristics and individual tip shape. Microgravity experiments aim at clarifying that dynamics, essentially free of convection, thus allowing identification of the main phenomena and key parameters that enter into play during diffusion-controlled growth pattern formation. Such experiments also contribute to the creation of a benchmark experimental database which is required for the improvement and validation of numerical simulations.

Two series of DECLIC-DSI microgravity experiments that are reported here were performed using dilute SCN-DC alloys of concentration well outside the coupled zone (0.24 and 0.46 wt%DC, respectively). The analysis of the first series of experiments shed light on complex thermal conditions within the experimental setup^39^. It was evidenced that heat transport, including latent heat rejection inside the sample, must be considered in analyses and numerical simulations. The (most commonly nonplanar) shape of the isotherms depends on V and the position along the adiabatic zone. Thus the problem cannot be described using the classical frozen-temperature approximation (stating that the thermal field is fully fixed externally). On this basis, a new modeling approach was developed, and a 3D phase-field model was coupled with a time-dependent calculation of the thermal diffusion in the adiabatic zone^86^. Taking such non-ideal thermal conditions into account was key to improved agreement between experimental and simulated pattern dynamics and characteristics. It will be highlighted below how far several factors of “non-ideal” solidification, not only unfrozen thermal fields and macroscopic interface curvature but also crystal orientation and polycrystallinity, intervene in the cellular and dendritic pattern dynamics. Such deviations from the minimal, diffusion-controlled growth model are intrinsically attached to the elaboration of materials by solidification in bulk configuration. It is imperative to identify and analyze their impacts in terms of microstructure characteristics and dynamics.

**Fig. 7:**
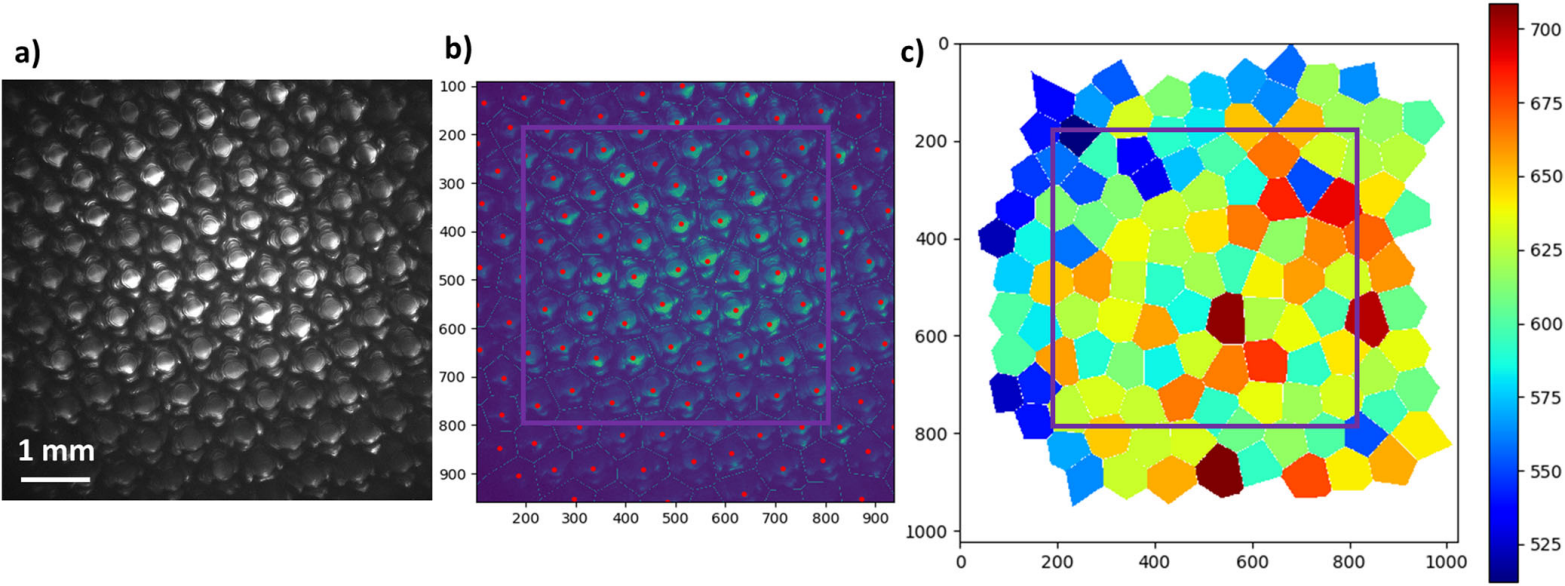
Dendritic patterns in DECLIC-DSI. In situ directional solidification of a SCN-0.46 wt% DC alloy (V = 1.5 μms^−1^; G = 12 Kcm^−1^) under microgravity (DECLIC-DSI). Raw images (**a**) are analyzed to determine each dendrite center and to build Voronoi skeletons (**b**) that are used to calculate local spacings (scale in µm) and draw maps of primary spacing (**c**)^89^. Color online.

It is well recognized that the shape of deep-cell and dendritic patterns is strongly influenced by the orientation of the growing crystal with respect to the main solidification axis and the isotherms^87,88^. In contrast, the consequences on large scales in spatially extended arrays in bulk samples remain essentially unexplored. In DECLIC-DSI experiments, great care has been taken to select single crystals with a [100] axis of the SCN crystal lattice (the growth axis of freely growing dendrites) parallel to z –to within a few degrees (< 3°). This led to unprecedented observations of large arrays of (essentially) axial cells and dendrites. The larger concentration of the alloy (SCN-0.46 wt%DC) resulted in well-developed dendritic patterns at rather low velocities (Fig. 7). We have developed several image analysis methods to achieve a robust identification of each dendrite position and size during solidification. Figure 7 illustrates the analysis process performed on a raw image to extract primary spacing values. In this example, a major effect of the macroscopic interface curvature on the primary spacing was identified (Fig. 2d)^89^. Such a macroscopic curvature tended to induce a radial drift of the pattern, which the anisotropic growth of dendrites resists. In addition, continual branching and elimination events prevented the formation of a well-ordered pattern in steady-state.

Cellular patterns with disordered hexagonal arrangement were formed at lower velocity for the less concentrated alloy^40^^,90^^,91^. Interestingly, not only steady-state but also oscillatory patterns were observed. A breathing-mode oscillation was identified, sometimes with a local coherence that is not generalized, however, over long distances due to the large-scale disorder of the pattern. An in-depth phase-field study^92^ led to a detailed description of the stability limits of steady-state and oscillatory patterns in a complete morphology diagram. The oscillations were also found to be sensitive to the misorientation of the crystal^91,93^. A criterion was proposed to estimate a critical misorientation angle above which oscillations are systematically damped out. In addition, it was possible to extract the complete cell tip shape evolution during the oscillation cycle from high-resolution interferometric images^40^. The shape variation is associated with an evolution of the concentration field, inaccessible experimentally but mediating the diffusive interactions between the cells. Full support was gained from 3D phase-field simulations that showed that transverse diffusive solute fluxes between neighboring cells are essential in the oscillation dynamics.

**Fig. 8:**
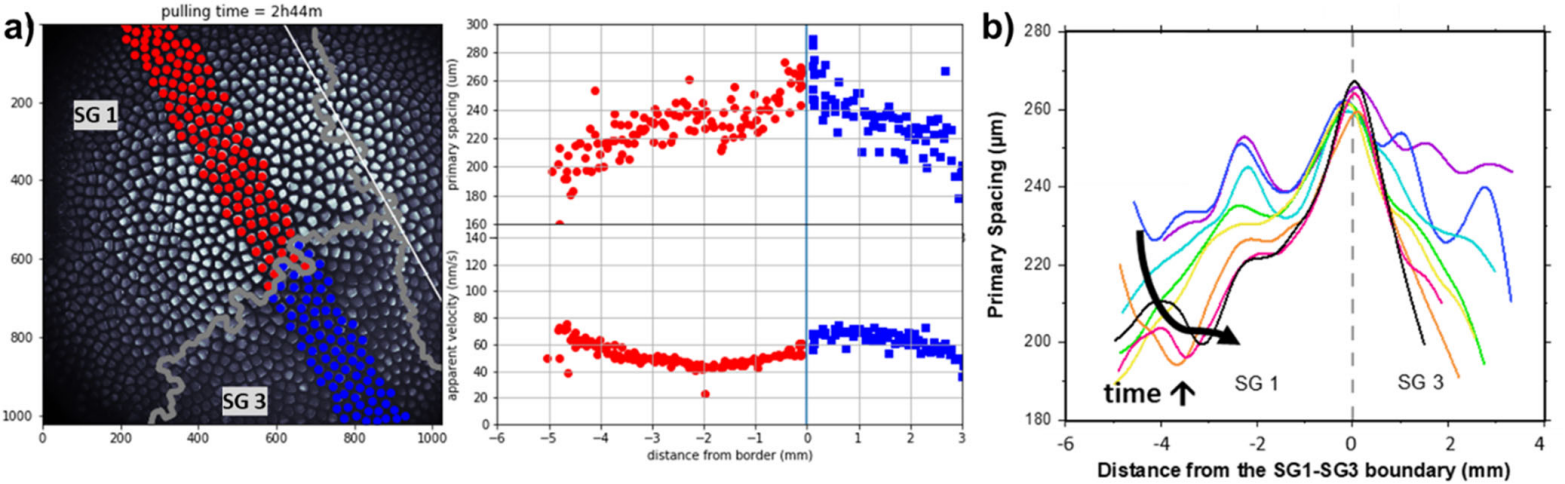
Drifting-cell grains. **a** Primary spacing and drifting velocity of the cells as a function of their distance from a divergent sub-boundary between two subgrains (SG1 and SG3, respectively), as indicated in the top-view image. **b** Time evolution of the spacing profile over 8.1 h (the experimental data points were fitted to smooth curves). Alloy: SCN-0.24 wt%DC. V = 2 μms^−1^. G = 19 Kcm^−1^. Color online.

A higher degree of complexity was induced in large single crystals containing a few low-misorientation grain boundaries. Those “sub-boundaries” delimit subgrains with slightly converging or diverging [100] crystal lattice axes—the ordinary growth direction of free dendrites (Fig. 8a). Due to the interfacial anisotropy, cellular patterns in non-axial grains drift laterally. Cell-spacing measurements revealed markedly different effects of divergent and convergent sub-boundaries on the spatiotemporal evolution of the patterns^94^. In brief, close to a divergent sub-boundary (Fig. 8b), the drifting motion of the cells away from each other induces a peaked, nonuniform spacing distribution that extends over typically twenty cell spacings. In contrast, a convergent sub-boundary induces a localized decrease of λ due to cell elimination.

On a larger scale, drifting cell patterns exhibited a complex spacing adjustment mechanism due to a sink-and-source dynamics near the container walls^84^. Ongoing analysis aims at a deeper understanding of the influence of sub-boundaries on the spatiotemporal organization of the array structure, as well as other dynamic phenomena such as sidebranching and the dependence of dendrite tip shapes on growth conditions.

Complex phenomena also occur along the sub-boundaries. In a recent work, Song et al. ^95^ report that during polycrystalline growth, a sub-boundary often adopts a wavy shape on the scale of λ (see Fig. 8a). A few cells belonging to one grain can eventually invade an adjacent grain, thus ending up with an intertwining of the two crystals. Systematic numerical simulations over a wide range of grain misorientations bring evidence for the general relevance of this experimental observation. Those results fundamentally alter the traditional view of well-separated cellular/dendritic grains in three dimensions.

### The columnar-to-equiaxed transition in dendritic growth

In metal alloy castings, columnar and equiaxed grains of the primary phase are common. In practice, columnar patterns designate dendritic arrays that usually grow during directional solidification, as they have been studied with the DECLIC-DSI. Equiaxed grains nucleate in the liquid or grow from detached primary-dendrite arms ahead of the main solidification front due to constitutional supercooling. The so-called Columnar to Equiaxed Transition (CET) describes a change in local grain structure that occurs in multicomponent alloys on a macroscale (the scale of the container) of a casting. It results in a textural change in the microstructure from elongated (columnar) to symmetrical (equiaxed) grain morphologies^1,2^. A complete CET occurs during transient conditions—varying external solidification conditions, changes of thermal and solutal fields—that favor the growth of equiaxed grains in a sufficient number and size so that the progression of the initial columnar dendritic front is blocked. In addition to the extension of the constitutionally supercooled liquid (and its evolution), important factors determining the CET are the nucleation rate and distribution of new crystals and/or the ability for solid fragments to detach from the columnar mushy zone and grow as equiaxed grains. Taking external conditions, nucleation and alloy parameters into account, Hunt^96^ proposed a simple criterion based on blocking fraction of equiaxed grains to derive a microstructure map depending on thermal gradient G and solidification velocity V. Explained simply, columnar grains are favored by intermediate to high-temperature gradients and slower growth rates. Equiaxed grains are favored by low-temperature gradients and higher solidification or growth rates. However, it is also known that convection in the liquid, and buoyancy effects as well, have a strong influence on the evolution of equiaxed grains (distribution of nucleation sites, motion of dendritic fragments). Obtaining reference observations with markedly reduced gravity effects is therefore key to gaining a better understanding of the CET dynamics.

Studies on CET in microgravity have been performed previously using metallic alloys^97^, where it was shown that a CET can occur either as a sharp or a progressive phenomenon. Modeling suggested that a progressive CET occurs by simultaneous columnar and equiaxed growth, the nucleation rate of equiaxed dendrites being an important factor. However, for obvious reasons, the analysis of microgravity experiments with the metallic system was performed by ex situ observations of cross-section micrographs. Model transparent alloy experiments offer the key advantage of in situ observation of the microstructure development, which, in the context of CET, is required to directly attest to the absence of convective transport or sedimentation/floatation of equiaxed grains.

During the CETSOL campaigns carried out onboard the ISS with the TA apparatus, the binary NPG-DC system within a hypoeutectic composition range, that is, mainly in the single-phased NPG region of the phase diagram, was used as a model alloy. The cartridges were of the same dimensions as for SEBA and SETA experiments, except for a substantially larger inner thickness (6 mm), making them suitable for the observation of nucleation and growth processes in a bulk container. Simply speaking, three groups of questions were defined, along with the design of dedicated experiments. The first one is related to the occurrence of columnar and equiaxed microstructure as a function of the thermal gradient, the pulling velocity and the alloy concentration (performed as part of the CETSOL-1 experiment). The second group of questions, to be addressed during future experiments (CETSOL-2), will be focused on obtaining observations close to casting conditions (that is, with a low-temperature gradient) but without convection. The third aspect concerns crystal orientation effects and will be investigated in detail in both experiments.

**Fig. 9:**
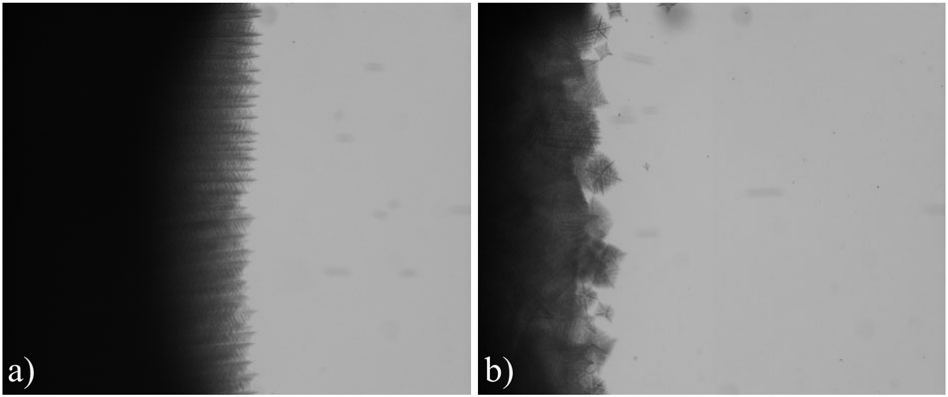
Columnar-to-equiaxed transition. Directional solidification of an NPG-30wt.%DC alloy in microgravity (TA apparatus). The pulling velocity was increased from 14 µms^−1^ (**a**) to 32 µms^−1^ (**b**). Vertical dimension of the images: 5.1 mm. In this figure, the images are presented with the solidification axis **z** (that is, the axis of the temperature gradient) horizontal (the liquid is on the right, the solid on the left).

During CETSOL-1, different values of the thermal gradient were applied, and stepwise increasing pulling velocities were imposed while observing the evolution of microstructures in a fixed field of view (side-view mode). The compositions of the NPG-DC alloys were chosen such that within the interval of three samples, a change of crystallographic orientation from <100> to <111> was expected^98^. Figure 9 shows a preliminary result from CETSOL-1. Here, a CET was induced by increasing the pulling velocity of the cartridge. The analysis of the ensemble of observations is an ongoing work.

During CETSOL-2 (to be performed tentatively in 2024), the central area of the sample will be placed at an (ideally) uniform temperature. The temperatures of the so-called hot and cold blocks will be identical, or a small temperature gradient will be applied. Starting with temperatures above the liquidus, thus from a uniform liquid, the system will be cooled down continuously to induce the growth of equiaxed dendritic grains (in isothermal mode). In situ observation of grain growth in bulk samples will provide the opportunity to characterize the multiple grain interactions that determine the final grain structure. Characterization of individual grains will also be performed (global grain shape, internal spacings, evolution and coarsening), and specific attention will be given to the selection of the dendritic growth direction as the chosen alloy compositions are similar to those of CETSOL-1. Attention will be paid to stereological corrections that are needed to account for the side-view projection imaging of the three-dimensional structures^99^. The experimental results will be compared to a volume-averaged model of equiaxed growth, including a suitable grain nucleation model^100^.

## Outlook and summary

It is well known and widely experienced within the scientific community that there is a long lapse of time between the moment when a microgravity experiment campaign in the ISS is defined and when it is actually carried out. As hopefully demonstrated above, this delay is actually useful to carry out in-depth on-ground experiments. This research has already yielded numerous important scientific results and has helped to refine the design of the experiments for an optimal use of the microgravity resources.

The need for solidification microgravity experiments with in situ diagnostics, including real-time imaging and monitoring, as well as telescience and remote control of experimental parameters, is evidenced. Technological innovation and transfer for the design of the TA and DECLIC-DSI instruments is also a key factor for obtaining accuracy and novelty. In this context, the long-term opportunity given by the European (ESA), American (NASA), French (CNES), German (DLR), and Austrian (FFG-ASAP) space agencies to the scientific partners for developing the research projects presented in this review has been highly beneficial.

Major scientific objectives that concern morphological transitions, interfacial-anisotropy effects, transient processes and nucleation-and-growth phenomena and their interaction with a competition between diffusion and convection during solidification have been reached or are about to be reached in the next few years. Many new phenomena have been observed directly with time and space resolutions that are allowed by the use of model transparent alloys. This research has had an impact on our fundamental understanding of nonequilibrium pattern formation and has increased our knowledge of engineering-directed aspects of great relevance for alloy casting and crystal growth. A major progress is shown with the quantitative match between carefully performed experimental observations and accurate numerical simulations and theoretical concepts. The increasing use of phase-field models and the upscaling of optimized simulation models are certainly some of the most salient outputs of solidification-in-microgravity research and opens interesting perspectives for the modeling of casting and advanced manufacturing processes.

New phenomena or long-standing questions are to be addressed, which could benefit from the accumulated knowledge in solidification—including rapid solidification, melting processes, the effect of impurities and trace elements, and the solidification of multicomponent alloys. This is of great importance, in full awareness of a growing need for innovation in energy-saving and recycling processes to be implemented in industry—on Earth or in space.

## Data Availability

Data sets relevant to the present study are available from the corresponding author upon reasonable request.

## Code availability

The code for phase-field simulations is available from the corresponding author upon request.
